# Stability of Properties of Layer-by-Layer Coated Membranes under Passage of Electric Current

**DOI:** 10.3390/polym14235172

**Published:** 2022-11-28

**Authors:** Ksenia Solonchenko, Olesya Rybalkina, Daria Chuprynina, Evgeniy Kirichenko, Ksenia Kirichenko, Victor Nikonenko

**Affiliations:** 1 Physical Chemistry Department, Kuban State University, 350040 Krasnodar, Russia; 2 Analytical Chemistry Department, Kuban State University, 350040 Krasnodar, Russia; 3 Department of Public and International Law, Kuban State Agrarian University named after I.T. Trubilin, 350004 Krasnodar, Russia

**Keywords:** layer-by-layer, ion exchange membrane, membrane modification, layer-by-layer stability, electrodialysis, monovalent selectivity, heterogeneous membrane, voltammetry, polyallylamine, polyethylenimine

## Abstract

Electrodialysis with layer-by-layer coated membranes is a promising method for the separation of monovalent and polyvalent ions. Since the separation selectivity is significantly reduced in the presence of defects in the multilayer system, the stability of the modifiers becomes an important issue. This article reports the i-V curves of layer-by-layer coated membranes based on the heterogeneous MK-40 membrane before and after 50 h long electrodialysis of a solution containing sodium and calcium ions at an underlimiting current density, and the values of concentrations of cations in the desalination chamber during electrodialysis. It is shown that the transport of bivalent ions through the modified membranes is reduced throughout the electrodialysis by about 50%, but the operation results in decreased resistance of the membrane modified with polyethylenimine, which may suggest damage to the modifying layer. Even after electrodialysis, the modified membrane demonstrated experimental limiting current densities higher than that of the substrate, and in case of the membrane modified with polyallylamine, the limiting current density 10% higher than that of the substrate membrane.

## 1. Introduction

The selective removal of monovalent ions from solutions is important for extraction of lithium from the brine of salt lakes [1,2] as well as for irrigation [3]. A simple method for creation of monovalent selective materials is the layer-by-layer assembly of a coating from polymers with alternating signs of the charges of polar groups [4,5,6].

The layer-by-layer assembly has been known since the 1960s, when it was used on oxides to create supports for chromatography [7]. The method reemerged in early1990s with inclusion of polymers [4] and with the use of direct surface force measurements [8]. The middle 1990s brought biomedical materials into consideration for the creation of layer-by-layer assembles [9]. Reviews of the biomedical applications of layer-by-layer assemblies are presented in [10,11].

The interest in layer-by-layer coating in the membrane separation field is related to the monovalent selectivity of the coating. Most promising was a study where a layer-by-layer coating of a perfluorosulfonic membrane increased its monovalent selectivity by several orders of magnitude [12]. An increase in selectivity, although more modest, was also reported under other conditions [13,14,15,16]. The layer-by-layer approach also allows creating monovalent selective anion exchange materials with reduced fouling [17]. A layer-by-layer coating is also used for reduction of fouling in other electromembrane processes, such as reverse electrodialysis [18]. A new development in layer-by-layer assembly of polymers on ion exchange membranes is the use of a pulsed electric field that allows faster formation of denser systems [19].

One of the most common ways to create a layer-by-layer coating is sequential adsorption from individual polymer solutions [20], and the most common mechanism for anchoring of a new layer is the electrostatic interaction of the polar groups of the new layer with the polar groups of the previous layer [21,22]. Emerging strategies of layer-by-layer assemblies, such as spin deposition, calcinations and dry-transfer printing, are discussed in [23]. By the electrostatic self-assembly mechanism, if the number of formed layers is small, a charge overcompensation occurs, and the adsorption step can be repeated [24,25].

Anchoring is considered reliable, since a large number of groups are bound in the electrostatic interaction at once, and the simultaneous dissociation of each of them is unlikely [26].

There is evidence that the absence of defects in the formed coating is extremely important for the selectivity of separation [27,28], and imperfections of real coatings or the accumulation of defects in the inner layers (for layer-by-layer assembly it can be trapped electrically neutral solution or layers mixed as a result of interdiffusion of polymers [29,30]) reduces the achieved selectivity.

A decrease in selectivity due to transport through defects has raised interest in the stability of layer-by-layer assembled coatings during operation. Some damage to the layers may occur in the assembly of the apparatus due to such events, such as partial drying and swelling of the membranes, changes in concentration of the solution, contact with o-rings, and possible bends. Other kinds of damage may occur during the operation. For example, electrodialysis membranes are affected by such factors as the passage of an electric current, contact with the components of the treated solution [31], heating [32], the presence of a tangential flow of solution, convection near the surface, and a change in pH that can discharge weakly acidic or weakly basic polar groups, transform quaternary ammonium bases into weakly basic amine groups [33] and decay the polymeric backbone containing polyvinyl chloride [34,35]. Research in [36] reports the properties of layer-by-layer self-assembled anion exchange membranes and shows that the sustained attack of from hydroxyl groups can cause the degradation of polymer skeleton and even breakdown of the membrane. An example of damage caused to a perfluorinated membrane by harsh conditions is given in article [37] describing the degradation of a perfluorinated membrane as a result of thermomechanical processes in a fuel cell. The factors occurring during electrodialysis can damage the polymer structure and wash its fragments away from the surface.

The present article describes how the barrier effect of coatings for transport of bivalent ion has changed, based on the analysis of concentrations in the desalination channel during electrodialysis desalination of a mixed solution containing monovalent and bivalent ions, and on i-V curves of layer-by-layer coated membranes recorded before and after the electrodialysis. It is shown that short-term operation does not lead to a noticeable decrease in the barrier effect of modified membranes, which would be expressed as a faster rate of decrease of concentration of bivalent ions later in electrodialysis compared with earlier in electrodialysis. However, the i-V curves registered before and after the electrodialysis differ and, in the case of a membrane modified with polyethylenimine, the membrane operation decreases the resistance of the system, which can be interpreted as the beginning of the loss of the barrier effect, which is not yet of a magnitude detectable on the kinetic dependences of concentration.

## 2. Materials and Methods

### 2.1. Membrane Modification

Commercially available heterogeneous MK-40 membrane (Shchekinoazot, Pervomaisky, Russia) was chosen as the substrate for the creation of modified membranes. The manufacturer claims that the membranes are produced by hot rolling of the powders of polyethylene and copolymer of sulfonated styrene and divinylbenzene between two polyamide cloths.

The technique for membrane modification by layer-by-layer coating is based on the technique described in [38], and was also used in our previous work [39]. The membranes were modified as follows.

The membranes were first swollen during stepwise equilibration with mixed solution with solutions containing equal equivalent concentrations of NaCl and CaCl_2_ with increasing dilution (the salts were purchased in solid form from Vekton, St. Petersburg, Russia; distilled water used here and below was produced in the laboratory), and some of these membranes were used later as controls. For other samples, to ensure the chemical uniformity of the surface, a homogenizing layer of LF-4SC perfluorinated sulfonic cation exchanger was applied to one side of the membranes by spreading a 0.5 mL of 7.2% (*w*/*w*) dispersion (purchased from Plastpolymer, St. Petersburg, Russia) over a 2 × 2 cm^2^ area in the center of a sample, then the samples were left for 30 min in air to evaporate the solvent. The monovalent selectivity and electrochemical properties of a bilayer membrane consisting of an MK-40 substrate and an LF-4SC modifying layer are described in [40].

For the next step, the three modifying layers of polymers were adsorbed on the LF-4SC layer at the membrane surface. Two series of modified membranes were created. For both series, the procedure consisted of 30 min of adsorption of polycation from 100 mL of 1 g/L dispersion, three rinses with distilled water, 30 min of adsorption of polyanion (sodium polystyrene sulfonate, PSS) from 100 mL of 1 g/L dispersion, three rinses with distilled water, 30 min of adsorption of the same polycation from new portion of 100 mL of 1 g/L dispersion, three rinses with water, and placement in the mixed solution with approximate concentration of salts of 15 mM NaCl + 7.5 mM CaCl_2_ (precise concentrations were later determined by chromatography) for 24 h. For the first series the polycation was polyallylamine (PAH), average molecular weight 70,000, purchased as a 30% aqueous solution from Sigma-Aldrich (now Merck KGaA, Darmstadt, Germany) and the polyanion was sodium polystyrene sulfonate (PSS, average molecular weight 15,000, purchased as a 20% aqueous solution from Sigma -Aldrich (Merck KGaA, Darmstadt, Germany). For the second series of modified membranes, the polycation was polyethylenimine (PEI, average molecular weight 25,000, purchased in solid form from Sigma-Aldrich (Merck KGaA, Darmstadt, Germany) and the polyanion was the same PSS as for the first series. The dependence of the properties of a membrane similar in composition to the LF-4SC polymer and modified with a polyamine on the method of preparation, is described in [41].

Thus, two series of experimental membranes were created. The first series had the MK-40–LF-4SC–PAA–PSS–PAA composition and was designated as MK-40-M (PAH), and the second series had the MK-40–LF-4SK–PEI–PSS–PEI composition and was later designated as MK-40-M (PEI).

**Figure 1:**
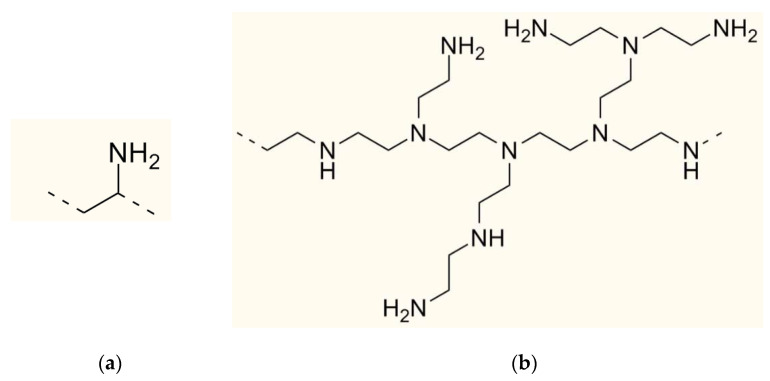
Elementary units of PAH (**a**) and PEI (**b**).

The structure of the PAH elementary unit and the approximate structure of the PEI elementary unit are shown in Figure 1.

From the structural point of view, PEI seems to be more promising for monopolar membrane modification since part of its ammonium content consists of tertiary amino groups that are less catalytically active in reaction of H^+^ and OH^−^ ions generation and they might cause a less undesirable shift of the pH of the treated solution. However, the PAH and the PEI also differ with respect to environmental and legal considerations. For example, if the membranes are modified for the treatment of irrigation water for greenhouse crops, further destined for ingestion, the use of PEI, which is assigned the Globally Harmonized System of Classification and Labelling of Chemicals P273 code, is not desirable. If the modifier is not produced in the country where the membranes are to be modified and to be used, it should be taken into account that polyethylenimine is classified as a dangerous good and requires special transportation conditions [42], while polyallyamine does not. Hence, it was also tested if the use of the less safe PEI would add any benefits in comparison with the safer PAH.

### 2.2. Voltammetry

To determine the electrochemical properties of the membranes, i-V curves of the membranes were recorded using a laboratory grade four-chamber flow-through electrodialysis cell. The procedure of registration of the i-V curves and the cell were the same as in our previous work [39]. The cell consisted of four chambers separated by the membranes (one studied membrane, one auxiliary MK-40 cation exchange membrane to separate the cathodic chamber and one auxiliary MA-41 anion exchange membrane from the same manufacturer to separate the anodic chamber) with a total area of 5 × 5 cm^2^, a polarized area of 2 × 2 cm^2^, and an intermembrane distance of 0.65 cm. The mixed solution of the same batch as the equilibrating solution contained approximately 15 mM NaCl and 7.5 mM of CaCl_2_ (the equivalent concentration was 15 mM ½ CaCl_2_) and was fed into all chambers at a linear rate of 4 mm/s by gravity from a common tank located above the cell, in which a constant liquid level was maintained by return of the solution from the cell by a Heidolph Pumpdrive 5001 peristaltic pump (Heidolph, Schwabach, Germany). The cell had two polarizing electrodes made of smooth platinum, and two Ag/AgCl reference electrodes. A Keithley 2200-60-2 power supply (Keithley Instruments, Cleveland, OH, USA) set the current sweep in the system stepwise in the range of current densities of 0–7.5 mA/cm^2^; the recording time of one i-V curve was 25 min. The potential drop at the membrane and the electric current density in the system were registered using a Keithley 2010 multimeter (Keithley Instruments, Cleveland, OH, USA). The values of pH were recorded in a common tank that fed all chambers, and in an outlet of desalination chamber.

### 2.3. Electrodialysis of Mixed Solution

**Figure 2:**
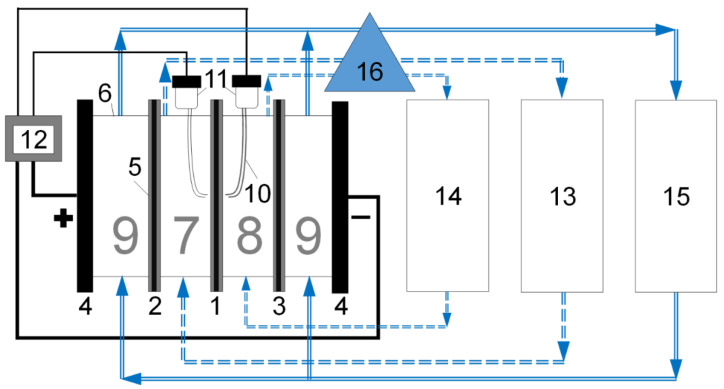
Scheme of the electrodialysis cell in the variant used for electrodialysis. The studied membrane is shown as 1, 2 and 3 are the auxiliary anion exchange and cation exchange membranes, respectively, 4 are the polarizing electrodes attached to the plexiglass plate, 5 is a rubber o-ring, 0.075 cm thick in assembled state, 6 is a plexiglass frame 0.5 cm thick, 7 is the desalination chamber, 8 is the concentration chamber, 9 are the electrode chambers, 10 is a Luggin capillary, 11 are the Ag/AgCl electrodes, 12 are the power source and a multimeter, 13 is the circulation tank of a desalination tract, 14 is the circulation tank of a concentration tract, 15 is the circulation tank of electrode tract, 16 is the multichannel pump. The figure is reproduced from our previous article [39].

After recording the first i-V curve, the desalination tract, the concentration tract and the electrode tracts were completely separated from each other as shown in Figure 2, so that a desalination tract was formed consisting of a desalination chamber, an auxiliary tank with a volume of at least 4 L and connecting tubes, a concentration tract was formed consisting of a concentration chamber, an auxiliary tank with a volume of at least 4 L and connecting tubes, and an electrode tract was formed consisting of two electrode chambers, an auxiliary tank with a volume of at least 4 L and connecting tubes.

After that, 4 L of the mixed solution was placed in tank of each of the tracts, the polarizing current of 1.5 mA/cm^2^ was turned on and the system continuously desalted the circulating mixed solution for 50 h. Every 10 h, 1 mL aliquots were taken from the tank in the desalination tract, and the concentration of Na^+^ and Ca^2+^ was determined using a Thermo Fisher Scientific Dionex Ultimate 3000 HPLC system (manufactured by Thermo Fisher Scientific, Sunnyvale, CA, USA) equipped with a Thermo Scientific Dionex IonPac CS16 column (manufactured by Thermo Fisher Scientific, Sunnyvale, CA, USA). It was assumed that the assembly and 50 h of operation would be enough for initial damage to the integrity of the applied layers would be noticeable.

After 50 h of electrodialysis, an i-V curve of a membrane was recorded by the procedure described above. Note that the experimental cell was not disassembled throughout the experiment.

### 2.4. Scanning Electron Microscopy

The layers formed on the surfaces of modified membranes were visualized using a JEOL JSM 7500F field emission scanning electron microscope (Jeol, 199 Tokyo, Japan) at the Collective Use Center “Diagnostics of the structure and properties of nanomaterials” of the Kuban State University. The samples were left in air to dry for 24 h, then cross sections were cut and visualizations of the cross sections produced. Images were made of two types of membranes: the first were membranes that were layer-by-layer coated, equilibrated with the mixed solution and then dried and cut without further treatments (i.e., these samples were the controls). The second type were membranes that were operated in electrodialysis (with registration of i-V curves prior and after the electrodialysis) and then taken from the experimental cell, dried and cut. The thickness of the coating was measured at four points for each sample.

## 3. Results and Discussion

### 3.1. Scanning Electron Microscopy

**Figure 3:**
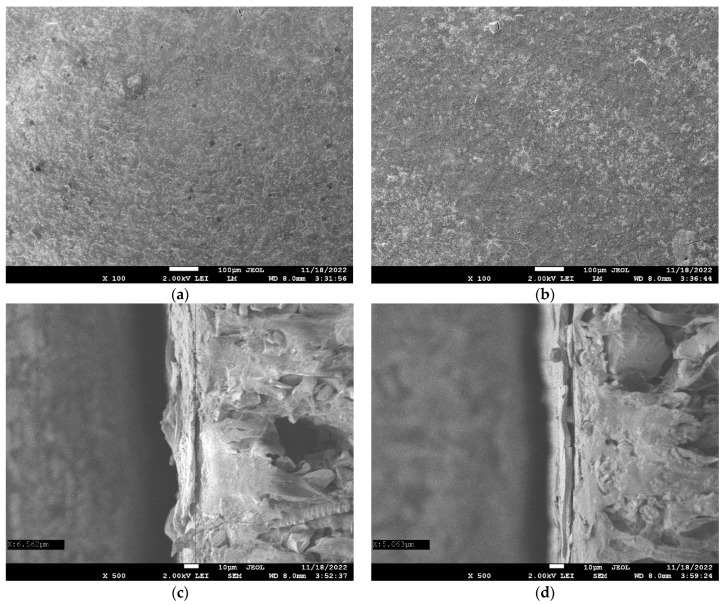
Top views (**a**,**b**) and cross sections of newly prepared MK-40-M (PAH) (**a**,**c**) and MK-40-M (PEI) (**b**,**d**) membranes imaged as dry samples.

Figure 3 shows images of the studied membranes obtained via scanning electron microscopy.

Top views of coating at MK-40-M (PAH) membrane revealed multiple dark dots, presumably defects of the coating, the diameter of which was about 10 μm. Top view of the coating of the MK-40-M (PEI) membrane was almost devoid of such dots, the sole candidate of which is visible in the bottom right corner of Figure 3b.

Determination of the thicknesses of coatings and their changes due to operation revealed that the thicknesses of the coating strongly depended on the coordinate, possibly due to filling of the relief of the substrate membrane; hence there are large confidence intervals. The obtained values were 5.12 ± 2.96 μm for newly prepared MK-40-M (PAH) membrane, 8.893 ± 7.25 μm for newly prepared MK-40-M (PAH) membrane, 7.75 ± 3.55 μm for MK-40-M (PAH) after electrodialysis and 4.13 ± 0.32 μm for MK-40-M (PEI) after electrodialysis. These values do not allow discarding the null hypothesis that all the thicknesses would be equal.

**Figure 4:**
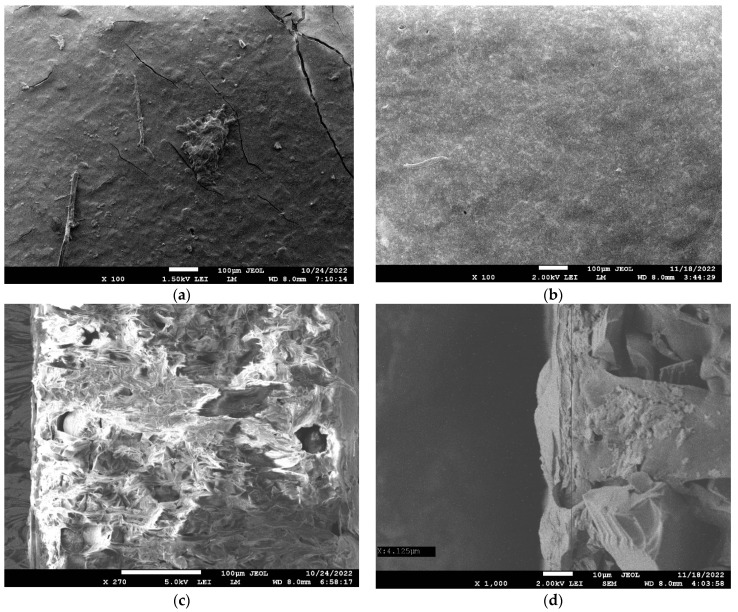
Top views (**a**,**b**) and cross sections of MK-40-M (PAH) (**a**,**c**) and MK-40-M (PEI) (**b**,**d**) membranes imaged as dry samples after their operation in electrodialysis.

After electrodialysis, dots appeared at the surface of MK-40-M (PEI) as well (Figure 4b), and the existing dots at the surface of MK-40-M (PAH) seemed to become more defined (circular caverns in Figure 4a, in which cracks are attributed not to membrane operation, but to drying prior to scanning electron microscopy), possibly indicating developing damage to the modifying layer.

### 3.2. i-V Curves before Electrodialysis

**Figure 5:**
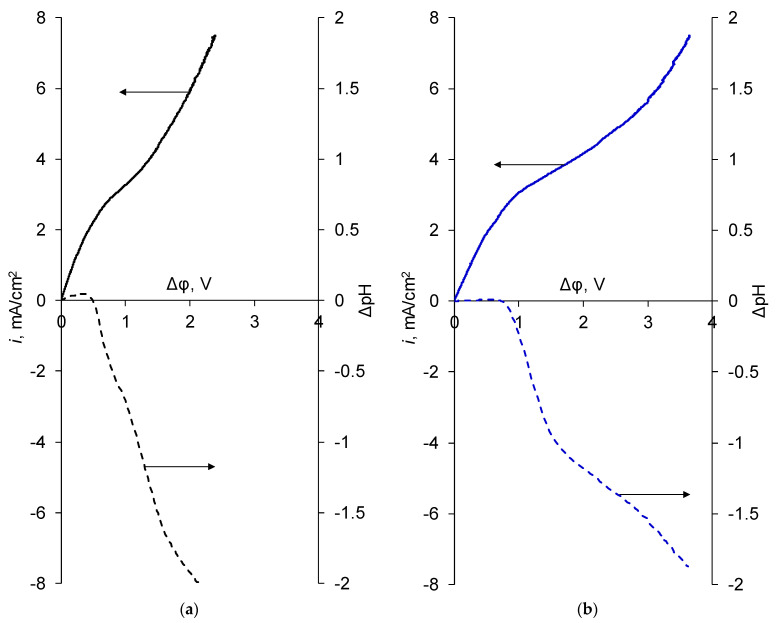

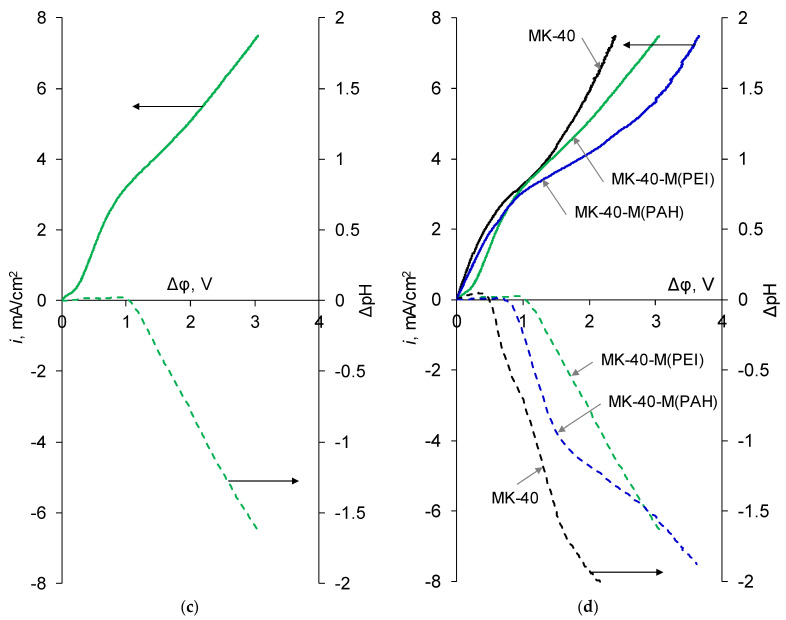
i-V curves recorded before electrodialysis of (**a**) MK-40, (**b**) MK-40-M (PAH) and (**c**) MK-40-M (PEI) membranes, as well as the pH difference between the output and the input into the desalination channel formed by the studied membrane and the auxiliary MA-41 anion exchange membrane. Panel (**d**) shows a comparison of all curves. Solid lines are i-V curves and dashed lines are pH curves.

Figure 5 shows the i-V curves of the membranes before the electrodialysis.

The shape of the i-V curve of the MK-40 membrane is typical for the i-V curve of a monopolar membrane [43]. It contains a so-called ohmic region (which contains not only ohmic contribution to the potential, but also the diffusion contribution to the potential, which also linearly increases with current density), a region of limiting current, which is somewhat diffuse as a result of membrane heterogeneity, and an unequal diameter of conductive regions on the membrane surface [44], a plateau region with a slope due to lateral diffusion of the electrolyte in the system [45], and a region of over-limiting currents.

The shape of the curve of the MK-40-M (PAH) membrane is similar to the shape of the i-V curve of the MK-40 membrane, except for a somewhat increased limiting current density (determined graphically by finding the intersection of tangents drawn to the so-called ohmic region and to the plateau) and a somewhat elongated plateau section. Growth of the limiting current is an advantage, since it allows using the higher currents and accelerating the desalination of solutions during electrodialysis in the galvanostatic mode. This growth can be caused both by the covering of nonconductive regions on the membrane surface by a layer of homogeneous LF-4SC material, which decreases the concentration polarization at the conductive regions [46], and by the possible appearance of regions containing polar groups of different signs of charge on the membrane surface (namely, the sulfonic groups of sodium polystyrene sulfonate and/or LF-4SC and the amino groups of PAH), which would cause the development of intensive electroconvection at the boundaries between these regions. The action of both of these mechanisms should lead to a reduction in the plateau region and an earlier transition to over-limiting currents, while an elongation of the plateau was experimentally observed. It is suggested that the emerging generation of H^+^ and OH^−^ ions plays a role in the lengthening of the plateau. Indeed, a noticeable divergence between the i-V curves of MK-40 and MK-40-M (PAH) membrane began with a potential drop at the membrane approximately equal to 1.2 V, and the turning point of the curve demonstrating the pH of the MK-40-M (PAH) membrane also occurred around this value. After 1.2 V was passed, the values of change in pH at the desalination channel with the MK-40-M (PAH) membrane were in a more alkaline region than those at the desalination channel with the MK-40 membrane. Taking into account the identical paired membrane in the channel, a change in pH in the alkaline region would mean that the generation of H^+^ and OH^–^ ions would be enhanced at the surface of the cation exchange membrane. The products of this reaction hinder the development of electroconvection, since they have a non-Stokesian mechanism of transport in solution themselves and, in addition, partially neutralize the extended space charge region [47,48]. The weakening of electroconvection can delay the transition to the region of over-limiting currents.

On the whole, the similarity of the shape of the current-voltage curve of the MK-40 and MK-40-M (PAH) membranes suggests that the transfer of ions through them occurs in a similar way.

The shape of the MK-40-M (PEI) membrane curve was significantly different. At the very beginning of the curve, there was a small segment of the rapid growth of the potential drop with a small increase in current density, which is more characteristic for the bipolar membranes [49]. The presence of this site can, as in the case of bipolar membranes, be explained by the barrier effect of the membrane to the transport of any salt ions. However, in contrast to classical bipolar membranes, this section was not long and it was replaced not by a zone of rapid increase in current density which occurs due to the intensive generation of H^+^ and OH^−^ ions, but by a region that resembled the so-called ohmic region of the i-V curves of monopolar membranes, followed by a region of limiting current. The limiting current density determined from the intersection of tangents drawn to the aforementioned so-called ohmic region and to the plateau region was 2.85 mA/cm^2^, which is close to the limiting current density of MK-40-M (PAH), which is 2.72 mA/cm^2^. An intensive generation of H^+^ and OH^−^ ions did not begin from the end of the region of rapid growth of potential drop, but began from the limiting current region. This means that the increase of the current occurring after the small initial section, and lasting at least until the limiting current density was reached, is caused by the transport of (mostly) cations of salts and not (solely) by generation and transport of H^+^ and OH^−^ ions. From this, we can conclude that the presence of layers formed on the membrane surface does not lead to complete blocking of ion transport and to transformation of the MK-40-M (PEI) into a bipolar membrane, but instead creates a potential barrier for such transport that is overcome with an increase in the potential drop.

The MK-40-M (PEI) also had increased generation of H^+^ and OH^−^ ions in comparison with the MK-40 membrane, a longer plateau region, and higher values of potential drop corresponding to the transition to over-limiting currents.

The observed differences between the membranes modified with different polymers were the motivation to compare the mass transport of monovalent and bivalent ions through such membranes, to investigate if the possible barrier effect of PEI layers hinders the transport of not only bivalent ions, which is the desired outcome, but also monovalent ions, which would hinder the performance of separation process, and to test the hypothesis that the PEI has a greater barrier effect to cation transport than the PAH and the use of PEI has advantage in comparison with use of PAH. To check this, the concentrations of ions during electrodialysis were measured.

### 3.3. Change of Concentrations during Electrodialysis

**Figure 6:**
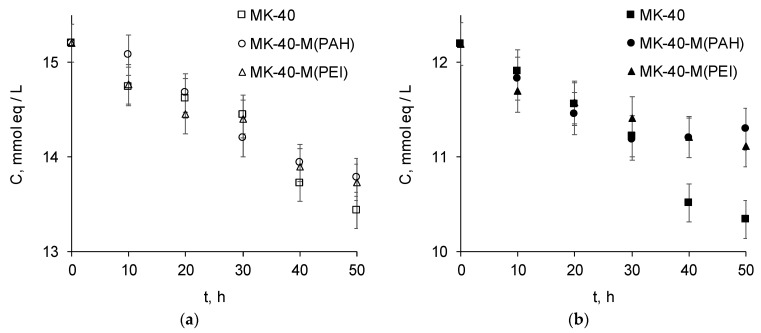
Concentrations of Na^+^ (**a**) and ½ Ca^2+^ (**b**) in the desalination tract during electrodialysis. Uncertainty margins reflect the uncertainties of chromatographic determination of concentrations.

Figure 6 demonstrates how the concentration of salt cations in the desalination tract during electrodialysis.

It can be seen that the Na^+^ concentration decreased for all tested membranes at the same rate. The concentrations determined for samples taken during electrodialysis with modified membranes tended to lie below the concentrations measured for the process with the original membrane, but this difference was within the uncertainty of the chromatographic determination of concentrations. In addition, the concentration of Na^+^ ions decreased at the same rate for both modified membranes. This does not confirm the hypothesis that the difference in the shape of the i-V curves of the modified membranes is due to the greater barrier effect of the MK-40-M (PEI) membrane for the transport of any cations.

The concentration of Ca^2+^ ions decreased significantly less during electrodialysis with the modified membranes than during electrodialysis with the commercial MK-40 membrane. If the ratio of ionic fluxes through the modified membranes to the flux through the substrate membrane is calculated as the ratio of the differences in Ca^2+^ concentration between the aliquots from 50 h of treatment and from 0 h of treatment, not taking into account the uncertainties of the chromatographic determination, then it can be seen that JCa^2+^ (mod)/JCa^2+^ (substrate) = ½. In addition, the difference in concentrations increased with time, so the results do not indicate the loss of selectivity of the modified membranes during electrodialysis.

Ideally the monovalent selective membranes should have a very high flux of monovalent ions, and a very small flux of polyvalent ions, corresponding to high permeability and high selectivity. In an ideal system there is always some sort of tradeoff. For example, here the modified membranes had a lesser flux of Ca^2+^ ions and an equal flux of Na^+^ ions compared with the commercial membrane, so they had lower permeability and higher monovalent selectivity. If the permeability was to become too low, then the use of such membranes would become economically unfeasible; however, in the considered case, at least the permeability for the monovalent ion was retained, making the operation of such a membrane comparable in cost with the operation of the commercial substrate membrane.

However, there were no significant differences between the two modified membranes, which additionally testified against the hypothesis of increased selectivity of the MK-40-M (PEI) membrane suggested on the basis of its i-V curve. It is possible that the barrier effect was present for the freshly prepared membrane but was lost during membrane operation. To check this suggestion, we considered the changes that occurred with the shape of the i-V curves of the studied membranes during their operation.

### 3.4. Changes in Shape of i-V Curves

**Figure 7:**
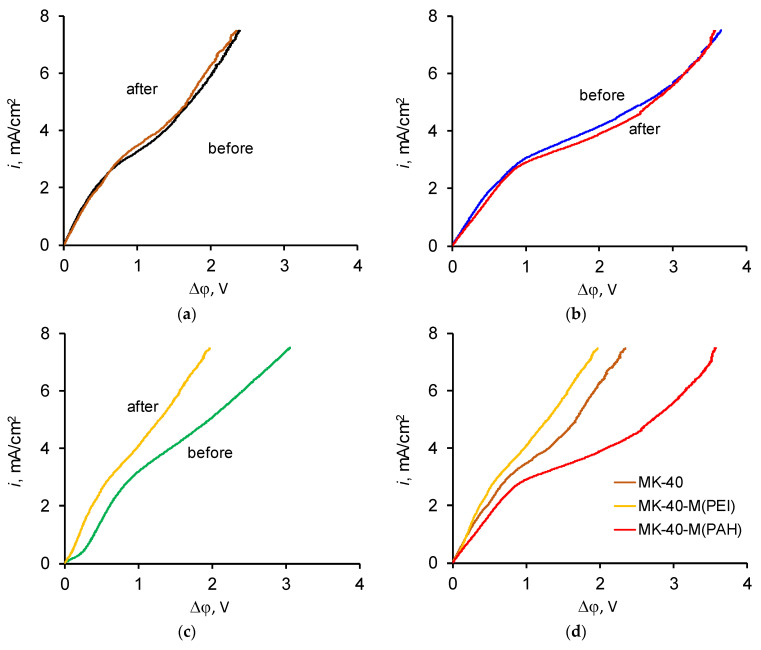
Pair-wise comparison of the i-V curves recorded before and after electrodialysis with (**a**) MK-40, (**b**) MK-40-M (PAH), (**c**) MK-40-M (PEI) membranes, as well as comparison of the i-V curves of all studied membranes after electrodialysis (**d**).

Comparisons of i-V curves of each membrane before and after operation are shown in Figure 7.

The i-V curves of MK-40 and MK-40-M (PAH) did not change very much, while the i-V curve of the MK-40-M (PEI) membrane changed greatly during operation of the membrane. The length of the initial section of the rapid growth of the potential drop in the i-V curve of this membrane significantly decreased, and the curve shifted towards lower potential drops at the same current densities. Limiting current densities, potential drops corresponding to limiting current densities, and changes in the average resistance of systems in the so-called ohmic section of i-V curves are given in Table 1.

Since the experimental cell was not disassembled during the experimental run, the changes cannot be attributed to the result of changing distances between the electrodes, and this shift must be attributed to the evolution of membrane properties. The shift to a lower potential drop may serve as evidence that the stronger barrier effect of MK-40-M (PEI) was present for the freshly prepared membrane but was lost during its operation. The recorded kinetic dependence of concentrations of Ca^2+^ in the desalination tract during electrodialysis with the modified membranes did not show a decrease in selectivity, which would prove the suggested hypothesis; a longer term study might provide more information. Indirect evidence of damage to the modifying layer was provided by the finding that the i-V curve of the MK-40-M (PEI) membrane after its operation was at lower potential drop range than the i-V curve of the MK-40 membrane after its operation, indicating additional mechanisms of mass transport. For now, the precise mechanism of the enhancement can only be postulated; it might be the removal of the adsorbed layers revealing the homogeneous layer of LF-4SC, which possesses better distribution of electric current lines over the surface, and it might be the revealing of some layer with polar groups charged oppositely to the charged groups of the topmost layer that produces electric heterogeneity and promotes the development of electroconvection.

From a practical aspect, a decrease in average membrane resistance can be considered an advantage, since an increased resistance means more energy consumption during electrodialysis in galvanostatic mode.

It is notable that the properties of the MK-40-M (PEI) membrane changed more strongly than the properties of the MK-40-M (PAH) membrane. Perhaps the reason for this is the nature of the polymer. The PEI used for modification is branched, and its main polymeric chains contain tertiary amino groups that can undergo chemical transformation, even cleaving under the action of the generated OH^−^ ions, leading to damage to the main polymer chains and the modifying layer. The reasons for the greater stability of the MK-40-M (PAH) membrane may be that it is linear, and its polar groups are represented only by primary amino groups, which are not necessary for the integrity of the main polymer chain and are capable of cleaving without damaging the overall structure.

The 50 h of operation was enough to observe some changes in the properties of the MK-40-M (PEI) membrane and to conclude that the coating of the MK-40-M (PAH) membrane was not damaged easily. Further studies of the stability of layer-by-layer coated membranes, and possibly studies of the ageing of such membranes during their lifetime operation over several years, would be of interest.

## 4. Conclusions

Layer-by-layer coating of heterogeneous MK-40 cation exchange membranes with polyallylamine and sodium polystyrene sulfonate in the first series, and polyethylenimine in the second series, created samples with higher monovalent selectivity than that of the substrate membrane. Determination of the concentrations of Na^+^ and Ca^2+^ ions during electrodialysis desalination of a mixed solution using the modified membranes showed that, in both cases, the transport of Na^+^ ions was within the margin of uncertainty as transport through the substrate membrane, but the transport of Ca^2+^ ions through the modified membranes decreased. The difference in transport of Ca^2+^ increased during the experiment, suggesting that the modified membranes retained increased monovalent selectivity during operation.

The experimental limiting current density of salt ions through the modified membranes exceeded the limiting current density through the commercially available membrane used as a substrate, which indicates that the new membranes had sufficiently high performance in the process of electrodialysis desalination. It was also shown that although the generation of H^+^ and OH^–^ ions at the modified membranes was greater than the generation at the substrate membrane, it became intensive only at over-limiting currents, meaning that the growth of the limiting current density was caused by a different mechanism, and that at the under-limiting currents the generation would not strongly affect the current efficiency or the purity of the product. At over-limiting currents, the generation of H^+^ and OH^–^ ions at the modified membranes became significantly greater, and the curves were shifted to a higher potential, which may suggest that the generation of H^+^ and OH^–^ ions suppressed the other mechanisms of mass transport, such as electroconvection.

The shape of the i-V curves of the modified membranes indicated that when the membranes were freshly modified, the cations were transported differently through the layers containing polyethylenimine and through the layers containing polyallylamine. The shape of the so-called ohmic region indicated that at low potential drops at the membrane (up to 0.3 V) there was a potential barrier to the transport of salt cations through the membrane modified with polyethylenimine, and the kinetic dependences of cation concentrations during electrodialysis suggested that this barrier affected the transport of Ca^2+^ more significantly. The electrical resistance of the system with the freshly prepared modified membrane containing polyethylenimine exceeded the electrical resistance of systems with the substrate membrane, or with the polyallylamine modified membranes.

If these differences persisted during operation, the increased electrical resistance would be a disadvantage; however, the i-V curve recorded after the membranes operated in electrodialysis showed a significant reduction in the length of this section. The over-limiting current region of the i-V curve of this membrane was also affected, shifting to lower potential drops. These changes might be attributed to damage to the modifying layers during operation.

Scanning electron microscopy showed that dark dots, presumably defects of the modifying layers, were present at the surface of the membrane modified with polyallylamine due to the modification, but were absent at the surface of the membrane modified with polyethylenimine. After electrodialysis, circular defects appeared at the surface of both membranes.

The i-V curves of the membrane modified with polyallylamine, and the substrate membranes, changed much less. The difference between the changes occurring with the curves recorded for modified membranes as a result of a fifty-hour long electrodialysis might be explained by the structure of polymers. Polyethylenimine contains several types of amino groups, including tertiary groups that may undergo transformation and even be cleaved under the influence of factors acting during electrodialysis, while polyallylamine contains only primary amino groups, the cleaving of which does not break the main chains.

## Figures and Tables

**Table 1:** Limiting current densities before and after the electrodialysis (ED), potential drops at the limiting current densities and the difference of average resistance at the so-called ohmic regions of i-V curves of the studied membranes.

Parameter	MK-40	MK-40-M(PAH)	MK-40-M(PEI)
*i*_lim_ before ED, mA/cm^2^	2.39	2.72	2.85
*i*_lim_ after ED, mA/cm^2^	2.48	2.75	2.50
Δφ at *i*_lim_ before ED, V	0.46	0.65	0.80
Δφ at *i*_lim_ after ED, V	0.49	0.83	0.44
*R*_Ohm after–_*R*_Ohm before_, Ohm	3.62	11.8	−58.1

## Data Availability

The data presented in this study are available on request from the corresponding author.
